# Rapid and facile one-step microwave synthesis of macrobicyclic cryptands

**DOI:** 10.1107/S2056989025003044

**Published:** 2025-04-29

**Authors:** Ulrich Baisch, Marie Christine Scicluna, Liana Vella-Zarb

**Affiliations:** a CrEMa Laboratories, University of Malta, Malta Life Sciences Park, San Gwann, SGN3000, Malta; Institute of Chemistry, Chinese Academy of Sciences

**Keywords:** crystal structure, liquid assisted grinding, cryptand, clathrochelate, microwave synthesis

## Abstract

Liquid-assisted grinding (LAG) and microwave synthesis are proposed as alternative routes for the synthesis of cryptands, with reaction times of up to 16 times faster than traditional methods. These rapid and facile techniques have the potential to replace traditional methods for a high-yield formation of clathrochelates, and other materials. The cryptand 6,16,25-tribenza-1,4,8,11,14,18,23,27-octa­aza­tri­cyclo­[9.9.9]nona­cosa-4,7,14,17,23,26-hexaene hexa­hydrate, C_36_H_42_N_8_·6H_2_O, (**Ph_3_T_2_**) was synthesized using this novel method. The crystal structure was redetermined by single-crystal X-ray diffraction using synchrotron radiation at 120 K. The structure exhibits disorder in the water mol­ecule of hydration.

## Abstract 2

Liquid-assisted grinding (LAG) and microwave synthesis are proposed as alternative routes for the synthesis of cryptands, with reaction times of up to 16 times faster than traditional methods.

## 1. Chemical context

Widely used in both academia and industry, microwave synthesis, an already established technique, is steamrolling to the forefront of organic synthesis. As opposed to the thermal conductivity and convection currents on which traditional forms of heating depend, microwave heating provides an inter­nal elevated-temperature system *via* efficient coupling of microwave energy with the mol­ecules in a reaction mixture. This markedly reduces reaction times to minutes, allowing for rapid synthesis and improving yields, thereby making large-scale synthesis possible (Kappe, 2008 ▸; Kappe *et al.*, 2009 ▸). Here we present the microwave-assisted synthetic technique as an easy yet powerful, energy-efficient replacement for the traditional wet chemical synthesis of crystalline Schiff-base clathro­chelate frameworks.
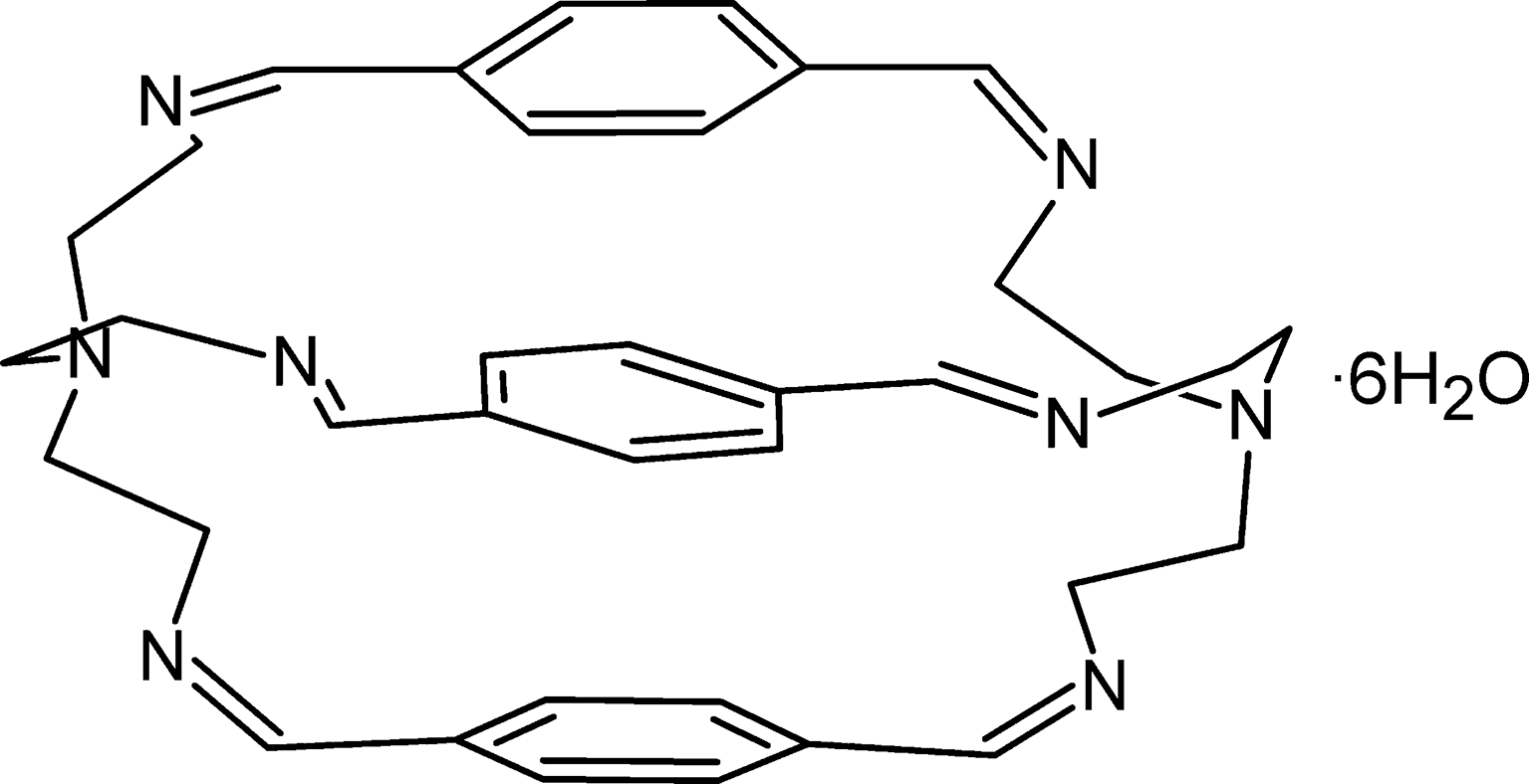


The hexa­imine macrobicycle **Ph_3_T_2_**, the simplified mol­ecular structure of which is shown in the scheme above, is used in particular as a host mol­ecule to co-encapsulate two silver or two copper(II) ions into a homodinuclear cage complex. Cryptands are widely exploited in hosting anion and cation guests, especially transition-metal ions (Youinou *et al.*, 1992 ▸; Drew *et al.*, 1992 ▸). They are also used as phase-transfer catalysts, transferring ionic reactants otherwise insoluble in organic solvents (Landini *et al.*, 1979 ▸), as ion-exchangers (Woodruff *et al.*, 2007 ▸), and as luminescence indicators for alkali ions (He *et al.*, 2001 ▸). The host–guest properties of clathrochelates further extend their utilization as precursors for the formation of mol­ecular Russian-doll superstructures (Cai *et al.*, 2018 ▸).

**Figure 1:**
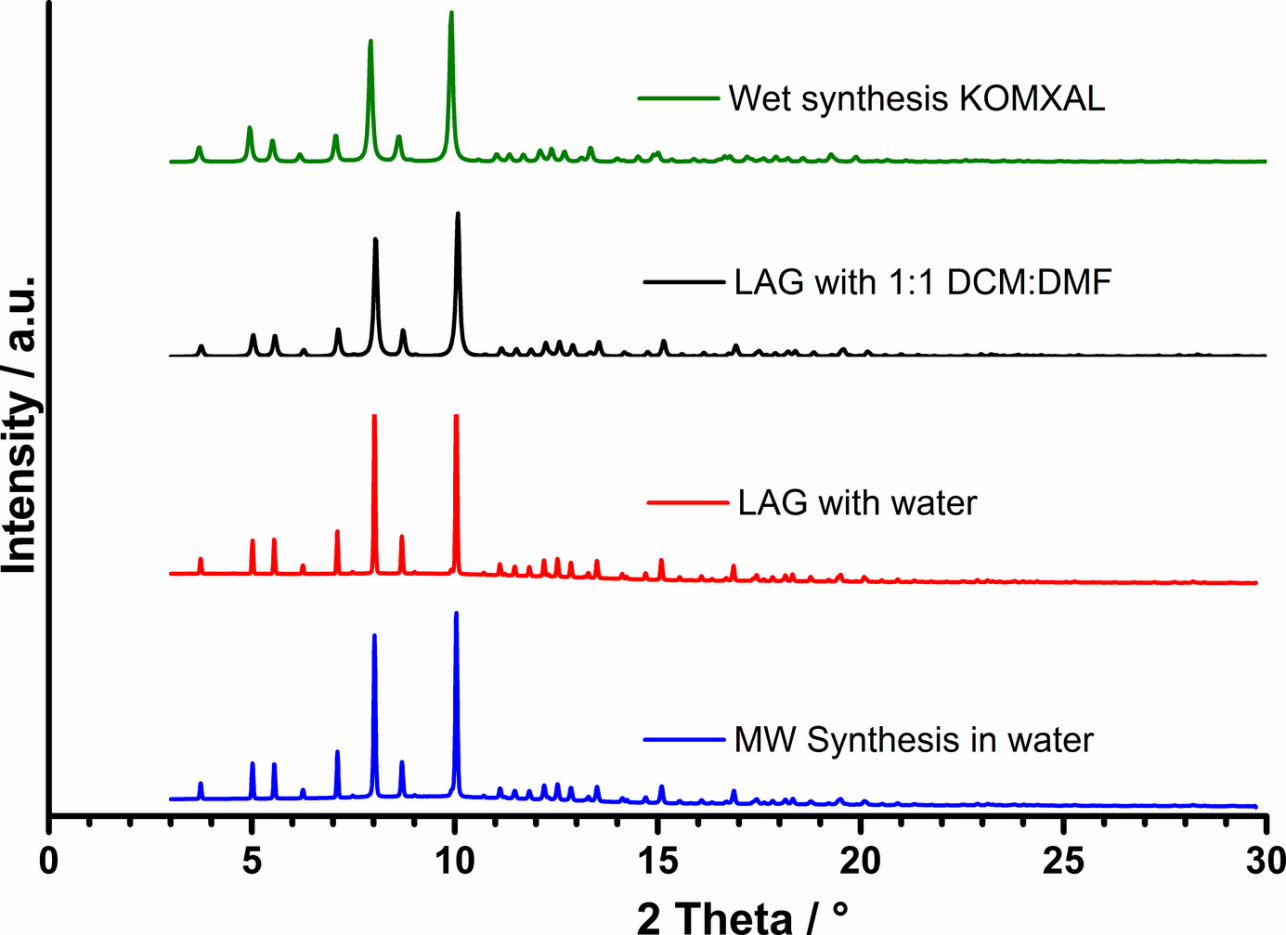
Powder diffraction patterns of cryptands obtained by: LAG with water (red); LAG with 1:1 DCM:DMF, calculated from synchrotron single-crystal data (black); MW synthesis in water (blue); and calculation from the SC data extracted from the CSD3 (green).

Open-vessel microwave processing, under atmospheric conditions, produced the same hexa­imine cryptand *via* 1 + 1 condensation of the synthons in 20 mL of water for 15 min at 150 W, followed by washing in acetone. This was again confirmed upon comparison with synchrotron data (Fig. 1 ▸). Both LAG and microwave techniques provided rapid routes for the successful synthesis of the imine macrobicyclic ligand, at an average reaction time which is more than 16 times faster than the 3–4 h required in wet chemical synthesis. As shown in Fig. 1 ▸, all three synthetic methods produced the same powder diffraction pattern. Laboratory powder diffraction data obtained for LAG synthesis in water solvent *via* 1 + 1 and 1 + 2 condensation ratios also matched the synchrotron data, leading to the observation that a change in molar ratio or in solvent mixture does not seem to affect the formation of the hexa­imine cryptand.

The LAG and microwave-assisted synthetic pathways of the ligands described in this study verify the feasibility of using these techniques to produce the same material as obtained *via* more traditional routes. This work demonstrates just one application of the green, rapid and facile techniques as an alternative to the synthesis of cryptands, which is often very expensive and quite difficult when compared to the synthesis of other ligands for alkali metals. However, by proof of concept this principle can pave the way for applications at large scales and in other fields.

## 2. Structural commentary

**Figure 2:**
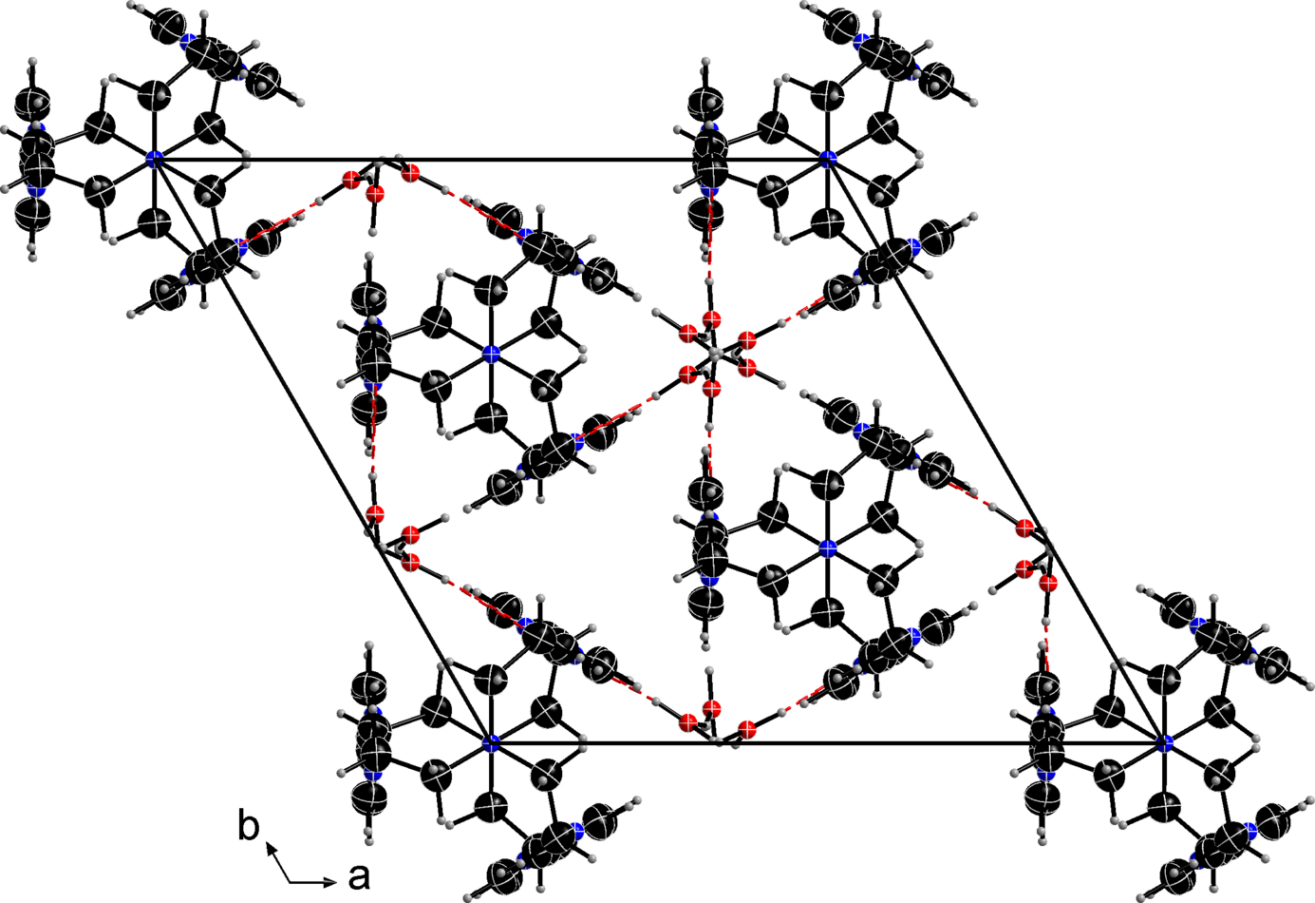
Crystal structure of **Ph_3_T_2_**. Displacement ellipsoids of all non-hydrogen atoms are drawn at the 70% probability level. Hydrogen bonds are drawn as red dashed lines.

**Figure 3:**
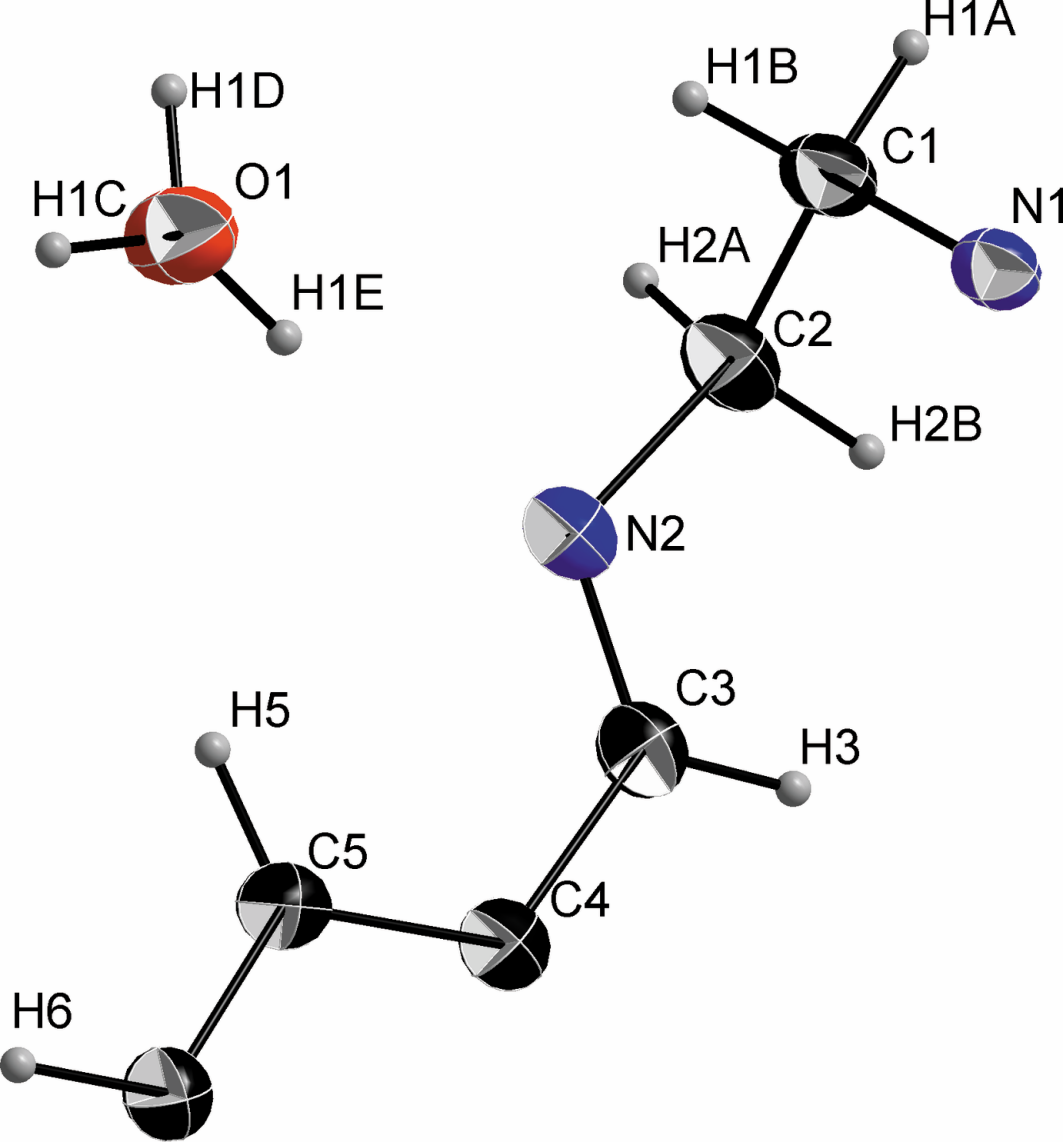
Asymmetric unit of the **Ph_3_T_2_** crystal structure with displacement ellipsoids drawn at the 50% probability level..

The crystal structure of **Ph_3_T_2_** consists of highly symmetrical discrete mol­ecular cages (cryptands), which are all oriented along the *c*-axis direction with respect to the central tertiary N atom (N1), the only atom on a special position in this structure). The asymmetric unit consists of 1/6 of the cage mol­ecule and one hydrate water mol­ecule. Thus, there are six hydrate water mol­ecules per **Ph_3_T_2_**. Fig. 2 ▸ shows the actual crystal structure in a projection down the cell axis *c* while Fig. 3 ▸ shows the asymmetric unit. The six water mol­ecules show disordered H atoms (with a fixed occupancy of 50% for H1*C* and H1*D*) are grouped closely around the threefold screw axis.

## 3. Supra­molecular features

Both the cryptand and the water mol­ecules of crystallization are strictly oriented and grouped along and around the threefold screw axis, forming chains (Fig. 2 ▸). The water mol­ecules seem to be the structure-forming element, each forming a hydrogen bond (Table 1 ▸) to a nitro­gen atom [O1⋯N2 = 2.857 (1)Å], to neighboring hydrate water mol­ecules [O1⋯O′1 = 2.807 (1) and 2.830 (1) Å], and one weak inter­action to C6 [C6⋯O1 = 3.517 (2) Å].

## 4. Database survey

All searches were carried out using the Cambridge Structural Database (CSD Version 2024.3.0; Groom *et al.*, 2016 ▸). A search for the structure of **Ph_3_T_2_** hexa­hydrate resulted in four hits. Only two report the corresponding silver (JOXJIP; Youinou *et al.*, 1992 ▸) and chromium (JAWSAB; Drew *et al.*, 1988 ▸) complexes, whereas the other two, report the same compound [KOMXAL (Drew *et al.*, 1992 ▸), KOMXAL01 (Zhu *et al.*, 2023 ▸)]. Both structures were determined from room temperature data from laboratory diffractometers, whereas the data for this work was acquired with synchrotron radiation at 120 K and thus, represents complementary data to the database.

Many hits can be found once the conjugated double bond to the phenyl rings between N2 and C3 is changed to a single bond. There are over 100 hits of cationic cryptands having a considerably different conformation reported in the database. This is due to a lack of a more extended π-electron system (*vide supra*).

## 5. Synthesis and crystallization

The template-free Schiff-base condensation of the tripodal amine *N*′,*N*′-bis­(2-amino­eth­yl)ethane-1,2-di­amine (TREN) with the dicarbonyl terephthalaldehyde generates the title hexa­imine binucleating macrobicyclic ligand. First isolated in 1992 (Drew, 1992 ▸), the free ligand was obtained as the hexa­hydrate *via* 2 + 3 condensation of the synthons stirred in 100 mL of aceto­nitrile for 3–4 h at ambient temperature and recrystallized from methanol.

In this study, the direct synthesis of the same Schiff-base capsule was carried out *via* 1 + 1 condensation of the synthons using two alternative synthetic techniques: liquid-assisted grinding (LAG) and microwave irradiation. In the first instance, for the mechanochemical synthesis, 0.5 ml of TREN and 0.4480 g of dialdehyde (1:1 molar ratio) were mechan­ically ground together using a Retsch MM400 ball mill using a catalytic amount of water (1 drop) for 12 min. Crystallization from 1.5 ml of water resulted in crystalline material. For the microwave synthesis, 0.4480 g of dialdehyde and 0.5 ml of TREN were added to 20 ml of deionised water and irradiated in a laboratory microwave for 2 min. Both the yellow crystalline powder resulting from the water-catalysed reaction, and the single crystals obtained from a 1:1 dichloromethane:dimethylformamide solvent-catalysed mixture were found to match the structure reported in the literature (*vide supra*), upon analysis of the corresponding synchrotron data (Fig. 1 ▸).

## 6. Refinement

Crystal data, data collection and structure refinement details are summarized in Table 2 ▸. H atoms were placed in calculated positions and refined as riding.

## Supplementary Material

Crystal structure: contains datablock(s) I. DOI: 10.1107/S2056989025003044/nx2023sup1.cif

Structure factors: contains datablock(s) I. DOI: 10.1107/S2056989025003044/nx2023Isup2.hkl

CCDC reference: 1889513

Additional supporting information:  crystallographic information; 3D view; checkCIF report

Additional supporting information:  crystallographic information; 3D view; checkCIF report

## Figures and Tables

**Table 1:** Hydrogen-bond geometry (Å, °)

*D*—H⋯*A*	*D*—H	H⋯*A*	*D*⋯*A*	*D*—H⋯*A*
O1—H1*C*⋯O1^i^	0.87	2.00	2.8075 (13)	154
O1—H1*D*⋯O1^ii^	0.87	2.03	2.8297 (13)	152
O1—H1*E*⋯N2	0.84 (2)	2.02 (2)	2.8566 (14)	170.3 (18)
C6—H6⋯O1^iii^	0.95	2.60	3.5173 (17)	163

Symmetry codes: (i) 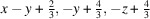; (ii) 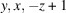; (iii) 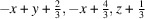.

**Table 2:** Experimental details

Crystal data	
Chemical formula	C_36_H_42_N_8_·6H_2_O
*M*_r_	694.87
Crystal system, space group	Trigonal, *R*32
Temperature (K)	120
*a*, *c* (Å)	14.5942 (2), 15.4565 (2)
*V* (Å^3^)	2851.03 (9)
*Z*	3
Radiation type	Synchrotron, λ = 0.64066 Å
μ (mm^−1^)	0.07
Crystal size (mm)	0.12 × 0.11 × 0.1

Data collection	
Diffractometer	Kappa CCD diffractometer at BM01 ESRF
Absorption correction	Multi-scan (*CrysAlis PRO*; Rigaku OD, 2018 ▸)
*T*_min_, *T*_max_	0.747, 1.000
No. of measured, independent and observed [*I* > 2σ(*I*)] reflections	4982, 1565, 1553
*R*_int_	0.017
(sin θ/λ)_max_ (Å^−1^)	0.683

Refinement	
*R*[*F*^2^ > 2σ(*F*^2^)], *wR*(*F*^2^), *S*	0.029, 0.082, 1.06
No. of reflections	1565
No. of parameters	83
H-atom treatment	H atoms treated by a mixture of independent and constrained refinement
Δρ_max_, Δρ_min_ (e Å^−3^)	0.26, −0.16
Absolute structure	Flack *x* determined using 691 quotients [(*I*^+^)−(*I*^−^)]/[(*I*^+^)+(*I*^−^)] (Parsons *et al.*, 2013 ▸)
Absolute structure parameter	0.2 (3)

Computer programs: *CrysAlis PRO* (Rigaku OD, 2018 ▸), *SHELXT* (Sheldrick, 2015*a* ▸), *SHELXL* (Sheldrick, 2015*b* ▸) and *OLEX2* (Dolomanov *et al.*, 2009 ▸).

## supplementary crystallographic information

### 6,16,25(1,4)-Tribenzena-1,4,8,11,14,18,23,27-octaazabicyclo[9.9.9]nonacosaphane-4,7,14,17,23,26-hexaene hexahydrate Crystal data

**Table:**

C_36_H_42_N_8_·6H_2_O	*D*_x_ = 1.214 Mg m^−^^3^
*M**_r_* = 694.87	Synchrotron radiation, λ = 0.64066 Å
Trigonal, *R*32	Cell parameters from 4384 reflections
*a* = 14.5942 (2) Å	θ = 1.9–26.0°
*c* = 15.4565 (2) Å	µ = 0.07 mm^−^^1^
*V* = 2851.03 (9) Å^3^	*T* = 120 K
*Z* = 3	Block, clear dark orange
*F*(000) = 1122	0.12 × 0.11 × 0.1 mm

### 6,16,25(1,4)-Tribenzena-1,4,8,11,14,18,23,27-octaazabicyclo[9.9.9]nonacosaphane-4,7,14,17,23,26-hexaene hexahydrate Data collection

**Table:**

Kappa CCD diffractometer at BM01 ESRF	1565 independent reflections
Radiation source: synchrotron	1553 reflections with *I* > 2σ(*I*)
Synchrotron monochromator	*R*_int_ = 0.017
ω scans	θ_max_ = 26.0°, θ_min_ = 1.9°
Absorption correction: multi-scan (CrysAlisPro; Rigaku OD, 2018)	*h* = −17→17
*T*_min_ = 0.747, *T*_max_ = 1.000	*k* = −19→19
4982 measured reflections	*l* = −19→19

### 6,16,25(1,4)-Tribenzena-1,4,8,11,14,18,23,27-octaazabicyclo[9.9.9]nonacosaphane-4,7,14,17,23,26-hexaene hexahydrate Refinement

**Table:**

Refinement on *F*^2^	Hydrogen site location: mixed
Least-squares matrix: full	H atoms treated by a mixture of independent and constrained refinement
*R*[*F*^2^ > 2σ(*F*^2^)] = 0.029	*w* = 1/[σ^2^(*F*_o_^2^) + (0.0592*P*)^2^ + 0.4923*P*] where *P* = (*F*_o_^2^ + 2*F*_c_^2^)/3
*wR*(*F*^2^) = 0.082	(Δ/σ)_max_ < 0.001
*S* = 1.06	Δρ_max_ = 0.26 e Å^−^^3^
1565 reflections	Δρ_min_ = −0.16 e Å^−^^3^
83 parameters	Absolute structure: Flack *x* determined using 691 quotients [(*I*^+^)-(*I*^-^)]/[(*I*^+^)+(*I*^-^)] (Parsons *et al.*, 2013)
0 restraints	Absolute structure parameter: 0.2 (3)
Primary atom site location: dual	

### 6,16,25(1,4)-Tribenzena-1,4,8,11,14,18,23,27-octaazabicyclo[9.9.9]nonacosaphane-4,7,14,17,23,26-hexaene hexahydrate Special details

**Table:**

Geometry. All e.s.d.s (except the e.s.d. in the dihedral angle between two l.s. planes) are estimated using the full covariance matrix. The cell e.s.d.s are taken into account individually in the estimation of e.s.d.s in distances, angles and torsion angles; correlations between e.s.d.s in cell parameters are only used when they are defined by crystal symmetry. An approximate (isotropic) treatment of cell e.s.d.s is used for estimating e.s.d.s involving l.s. planes.
Refinement. none

### 6,16,25(1,4)-Tribenzena-1,4,8,11,14,18,23,27-octaazabicyclo[9.9.9]nonacosaphane-4,7,14,17,23,26-hexaene hexahydrate Fractional atomic coordinates and isotropic or equivalent isotropic displacement parameters (Å^2^)

**Table:**

	*x*	*y*	*z*	*U*_iso_*/*U*_eq_	Occ. (<1)
O1	0.63158 (9)	0.60810 (8)	0.58950 (6)	0.0393 (3)	
H1C	0.662121	0.660324	0.625960	0.059*	0.5
H1D	0.636344	0.638318	0.539870	0.059*	0.5
N1	0.666667	0.333333	0.49411 (11)	0.0261 (3)	
N2	0.51572 (8)	0.38221 (8)	0.59508 (6)	0.0298 (2)	
C1	0.61250 (11)	0.38827 (10)	0.46282 (7)	0.0291 (3)	
H1A	0.602840	0.377965	0.399467	0.035*	
H1B	0.658443	0.465002	0.473532	0.035*	
C6	0.53551 (10)	0.43279 (10)	0.87019 (8)	0.0318 (3)	
H6	0.569410	0.501297	0.895476	0.038*	
C5	0.52976 (10)	0.42258 (9)	0.78113 (8)	0.0321 (3)	
H5	0.558765	0.483953	0.745679	0.039*	
C3	0.47781 (10)	0.30720 (9)	0.64941 (8)	0.0291 (3)	
H3	0.444964	0.236743	0.628129	0.035*	
C4	0.48177 (9)	0.32301 (10)	0.74337 (8)	0.0277 (3)	
C2	0.50528 (10)	0.35225 (11)	0.50410 (8)	0.0315 (3)	
H2A	0.469113	0.384344	0.472734	0.038*	
H2B	0.461084	0.274407	0.499024	0.038*	
H1E	0.5950 (16)	0.5422 (18)	0.5971 (12)	0.045 (5)*	

### 6,16,25(1,4)-Tribenzena-1,4,8,11,14,18,23,27-octaazabicyclo[9.9.9]nonacosaphane-4,7,14,17,23,26-hexaene hexahydrate Atomic displacement parameters (Å^2^)

**Table:**

	*U*^11^	*U*^22^	*U*^33^	*U*^12^	*U*^13^	*U*^23^
O1	0.0441 (6)	0.0328 (5)	0.0309 (5)	0.0116 (4)	−0.0030 (4)	0.0005 (3)
N1	0.0299 (5)	0.0299 (5)	0.0186 (7)	0.0150 (3)	0.000	0.000
N2	0.0312 (5)	0.0343 (5)	0.0251 (5)	0.0173 (4)	0.0015 (4)	−0.0017 (4)
C1	0.0348 (6)	0.0356 (6)	0.0197 (5)	0.0197 (5)	0.0015 (4)	0.0024 (4)
C6	0.0366 (6)	0.0275 (5)	0.0286 (6)	0.0140 (5)	0.0029 (5)	−0.0022 (4)
C5	0.0378 (6)	0.0281 (5)	0.0275 (6)	0.0144 (5)	0.0050 (5)	0.0018 (4)
C3	0.0292 (6)	0.0312 (5)	0.0269 (6)	0.0151 (5)	−0.0013 (4)	−0.0033 (4)
C4	0.0277 (5)	0.0302 (5)	0.0259 (5)	0.0151 (4)	−0.0002 (4)	−0.0009 (4)
C2	0.0336 (6)	0.0400 (6)	0.0237 (5)	0.0205 (5)	−0.0014 (4)	−0.0029 (4)

### 6,16,25(1,4)-Tribenzena-1,4,8,11,14,18,23,27-octaazabicyclo[9.9.9]nonacosaphane-4,7,14,17,23,26-hexaene hexahydrate Geometric parameters (Å, º)

**Table:**

O1—H1C	0.8703	C1—C2	1.5197 (18)
O1—H1D	0.8701	C6—H6	0.9500
O1—H1E	0.84 (2)	C6—C5	1.3826 (17)
N1—C1^i^	1.4614 (13)	C6—C4^iii^	1.3940 (17)
N1—C1	1.4614 (13)	C5—H5	0.9500
N1—C1^ii^	1.4614 (13)	C5—C4	1.3875 (17)
N2—C3	1.2665 (16)	C3—H3	0.9500
N2—C2	1.4578 (15)	C3—C4	1.4670 (16)
C1—H1A	0.9900	C2—H2A	0.9900
C1—H1B	0.9900	C2—H2B	0.9900
			
H1C—O1—H1D	104.5	C6—C5—H5	119.9
H1C—O1—H1E	131.6	C6—C5—C4	120.22 (11)
H1D—O1—H1E	123.1	C4—C5—H5	119.9
C1^ii^—N1—C1^i^	109.61 (8)	N2—C3—H3	118.2
C1^i^—N1—C1	109.62 (8)	N2—C3—C4	123.69 (11)
C1^ii^—N1—C1	109.62 (8)	C4—C3—H3	118.2
C3—N2—C2	116.45 (11)	C6^iii^—C4—C3	118.24 (11)
N1—C1—H1A	108.6	C5—C4—C6^iii^	119.02 (11)
N1—C1—H1B	108.6	C5—C4—C3	122.73 (11)
N1—C1—C2	114.65 (10)	N2—C2—C1	111.47 (10)
H1A—C1—H1B	107.6	N2—C2—H2A	109.3
C2—C1—H1A	108.6	N2—C2—H2B	109.3
C2—C1—H1B	108.6	C1—C2—H2A	109.3
C5—C6—H6	119.6	C1—C2—H2B	109.3
C5—C6—C4^iii^	120.73 (11)	H2A—C2—H2B	108.0
C4^iii^—C6—H6	119.6		

Symmetry codes: (i) −*x*+*y*+1, −*x*+1, *z*; (ii) −*y*+1, *x*−*y*, *z*; (iii) *x*−*y*+1/3, −*y*+2/3, −*z*+5/3.

### 6,16,25(1,4)-Tribenzena-1,4,8,11,14,18,23,27-octaazabicyclo[9.9.9]nonacosaphane-4,7,14,17,23,26-hexaene hexahydrate Hydrogen-bond geometry (Å, º)

**Table:**

*D*—H···*A*	*D*—H	H···*A*	*D*···*A*	*D*—H···*A*
O1—H1*C*···O1^iv^	0.87	2.00	2.8075 (13)	154
O1—H1*D*···O1^v^	0.87	2.03	2.8297 (13)	152
O1—H1*E*···N2	0.84 (2)	2.02 (2)	2.8566 (14)	170.3 (18)
C6—H6···O1^vi^	0.95	2.60	3.5173 (17)	163

Symmetry codes: (iv) *x*−*y*+2/3, −*y*+4/3, −*z*+4/3; (v) *y*, *x*, −*z*+1; (vi) −*x*+*y*+2/3, −*x*+4/3, *z*+1/3.
